# Detection and analysis of spatiotemporal patterns in brain activity

**DOI:** 10.1371/journal.pcbi.1006643

**Published:** 2018-12-03

**Authors:** Rory G. Townsend, Pulin Gong

**Affiliations:** 1 School of Physics, The University of Sydney, NSW, Australia; 2 ARC Centre of Excellence for Integrative Brain Function, The University of Sydney, NSW, Australia; University College London, UNITED KINGDOM

## Abstract

There is growing evidence that population-level brain activity is often organized into propagating waves that are structured in both space and time. Such spatiotemporal patterns have been linked to brain function and observed across multiple recording methodologies and scales. The ability to detect and analyze these patterns is thus essential for understanding the working mechanisms of neural circuits. Here we present a mathematical and computational framework for the identification and analysis of multiple classes of wave patterns in neural population-level recordings. By drawing a conceptual link between spatiotemporal patterns found in the brain and coherent structures such as vortices found in turbulent flows, we introduce velocity vector fields to characterize neural population activity. These vector fields are calculated for both phase and amplitude of oscillatory neural signals by adapting optical flow estimation methods from the field of computer vision. Based on these velocity vector fields, we then introduce order parameters and critical point analysis to detect and characterize a diverse range of propagating wave patterns, including planar waves, sources, sinks, spiral waves, and saddle patterns. We also introduce a novel vector field decomposition method that extracts the dominant spatiotemporal structures in a recording. This enables neural data to be represented by the activity of a small number of independent spatiotemporal modes, providing an alternative to existing dimensionality reduction techniques which separate space and time components. We demonstrate the capabilities of the framework and toolbox with simulated data, local field potentials from marmoset visual cortex and optical voltage recordings from whole mouse cortex, and we show that pattern dynamics are non-random and are modulated by the presence of visual stimuli. These methods are implemented in a MATLAB toolbox, which is freely available under an open-source licensing agreement.

## Author summary

Structured activity such as propagating wave patterns at the level of neural circuits can arise from highly variable firing activity of individual neurons. This property makes the brain, a quintessential example of a complex system, analogous to other complex physical systems such as turbulent fluids, in which structured patterns like vortices similarly emerge from molecules that behave irregularly. In this study, by uniquely adapting techniques for the identification of coherent structures in fluid turbulence, we develop new analytical and computational methods for the reliable detection of a diverse range of propagating wave patterns in large-scale neural recordings, for comprehensive analysis and visualization of these patterns, and for analysis of their dominant spatiotemporal modes. We demonstrate that these methods can be used to uncover the essential spatiotemporal properties of neural population activity recorded by different modalities, thus offering new insights into understanding the working mechanisms of neural systems.

This is a *PLOS Computational Biology* Methods paper.

## Introduction

Recent advances in brain recording techniques have led to a rapid influx of high spatial- and temporal-resolution datasets of large neural populations [1–4]. One of the major challenges in modern neuroscience is to identify and extract important population-level structures and dynamics from these datasets [5,6]. Traditionally, neural population activity has been mainly studied from the perspective of temporal synchrony or correlation, and relating correlated neural activity to brain functions has been the major focus of many studies in neuroscience during the past two decades [7,8].

However, growing evidence indicates that population-level brain activity is often organized into patterns that are structured in both space and time. Such spatiotemporal patterns, including planar traveling waves [9–11], spiral waves which rotate around a central point [12–14], source and sink patterns which expand or contract from a point [13,15], and saddle patterns which are formed by the interaction of multiple waves [13], have been observed at different neural levels within multiple recording techniques, including multi-electrode arrays [13,16–18], voltage sensitive dye (VSD) imaging [9,12,19], and electroencephalography (EEG), electrocorticography (ECoG), magnetoencephalography (MEG) and functional magnetic resonance imaging (fMRI) [20–24].

The functional role of these spatiotemporal patterns is a subject of active research: In spontaneous activity, propagating patterns have been shown to follow repeated temporal motifs instead of occurring randomly [13,15], and are postulated to facilitate information transfer across brain regions [10,17] and carry out distributed dynamical computation [25]. In sensory cortices, stimuli can elicit repeatable propagating patterns [9,10,19,26,27], and the properties of these waves can be linked to stimulus features. For instance, the phase and amplitude of traveling waves in the motor cortex and visual cortex correlate with reach target location [17] and with saccade size [18], respectively, and the propagation direction of moving patterns in the visual cortex is sensitive to visual movement orientation [28]. These studies thus indicate that the ability to detect and analyze these patterns is essential for uncovering the principled dynamics of neural population activity and for understanding the working mechanisms of neural circuits [15,26,29,30].

In this study, to detect changes of neural signals happening across both space and time, we introduce velocity vector fields which represent the speed and direction of local spatiotemporal propagations. These vector fields allow us to make a novel conceptual link between spatiotemporal patterns in neural activity and complex patterns such as vortices or eddies found in the field of fluid turbulence [31–33], in which these patterns are similarly characterized by using velocity fields of the underlying moving molecules. Velocity vector fields in our methods are computed by adapting optical flow estimation methods originally developed in the field of computer vision [34]. Optical flow techniques have previously been implemented to analyze brain activity [13,26–28], but here we extend these methods to consider the amplitude and phase of oscillatory neural signals, allowing for a comprehensive analysis of neural spatiotemporal patterns. When constructed from oscillation phase, velocity vector fields are conceptually similar to phase gradient vector fields as often used in previous studies [15,18]. However, velocity vector fields provide a conceptual basis for us to adapt methods from turbulence to develop a unified methodological framework for analyzing neural spatiotemporal patterns.

We show that by examining the critical points in a velocity vector field (also called “stationary points” or “singularity points”), where the local velocity is zero [35], different types of spatiotemporal patterns including spiral waves (“foci”), source/sink patterns (“nodes”) and saddles can be detected. In addition to these complex wave patterns, neural systems can exhibit widespread synchrony and planar travelling waves. These types of activity are common to many physical and biological systems, and can be detected by introducing global order parameters calculated from velocity vector fields [36]. These methods thus enable the automatic detection of a diverse range of spatiotemporal patterns after user-defined parameters have been chosen; these parameters are discussed in detail in Methods and Materials.

Aside from detecting these patterns, our methods can provide systematic analysis of pattern dynamics including their evolution pathways and their underlying spatiotemporal modes that exhibit intrinsic and inseparable spatial and temporal features, thus providing a novel alternative to existing dimensionality reduction techniques which instead separate space and time components [6]. We validate the effectiveness of all methods and their implementation in the toolbox through multiple approaches. Using synthetic data with known pattern activity, we show that spatiotemporal pattern detection is accurate and reliable even in noisy conditions. We then analyze local field potentials from multi-electrode arrays in marmoset visual cortex and whole-brain optical imaging data from mouse cortex to test our methodological framework across different recording modalities, species, and neural scales. We find that pattern properties including location and propagation direction are modulated by visual stimulus, and that patterns evolve along structured pathways following preferred transitions.

## Results

Here we outline a methodological framework for detecting and analyzing wave patterns in neural recordings using velocity vector fields. These methods can be applied to any recording methodology with high spatial and temporal resolution, including multi-electrode LFPs, VSD and optical imaging, ECoG, EEG, and MEG. Some of these methods have been briefly described in our previous work [13], but in this paper we examine them in more depth and show how they can be uniquely combined with new techniques to form a systematic framework for pattern detection and analysis. We also discuss their implementation into a freely available MATLAB toolbox, the NeuroPatt toolbox. Neural data from a two-dimensional spatiotemporal recording are represented by a four-dimensional matrix, $z\left(x,y,t,p\right)$, where *z* is the recorded signal with regular spatial coordinates $\left(x,y\right)$, time *t*, and trial presentation *p*. Recordings without repeated trials or averaged across all trials have p=1, although we caution that trial-averaged signals typically do not capture the spatiotemporal patterns present in single trials [24,28].

### Velocity vector fields

Neural activity, although appearing highly disordered at the single-neuron level, can form dynamical coherent structures such as propagating waves at the population level [37]. There are many other complex systems that display similar emergent pattern dynamics, including fluid turbulence, in which coherent flows and vortices emerge from interacting molecules that behave irregularly [31,32]. Velocity vector fields, which represent the direction and speed of fluid motion, are essential mathematical tools for detecting and analyzing coherent activity patterns embedded in turbulent flow [32]; studies using this approach typically separate activity patterns at different scales, independently detecting both large-scale flows and small-scale eddies [33]. In turbulent flow, velocity vector fields are typically measured by following the movement of tracer particles within the fluid [38]. Here, we introduce a method for calculating analogous velocity vector fields in neural signals, representing the local direction and speed of propagating activity at each recording site. As for in studies of coherent structures in turbulence, these velocity fields obtained in neural data provide a powerful conceptual framework for analyzing a diverse range of propagating wave patterns in the brain.

For a data sequence *D(x*,*y*,*t)*, which may represent the raw recorded signal *z* or the amplitude *A* or phase *θ* of an oscillatory neural signal, extracted by using either the Hilbert transform [39] or complex Morlet wavelets [40] (see Oscillatory data filtering in Methods and Materials), the velocity vector field w(x,y,t)=(u(x,y,t),v(x,y,t)) represents the velocity in *x*- and *y*-directions at each location between time *t* and *t+δt*, where *δt* is the time step specified by the sampling frequency. If data contain multiple trials, the velocity vector field is computed iteratively for each trial.

To calculate the velocity field *w(x*,*y*,*t)*, we adapt optical flow estimation techniques from the field of computer science, which were first developed by Horn and Shunck to track the motion between successive frames of a sequence of images [34]. In their original formulation, the optical flow is calculated by solving two constraints. The first is the data constancy constraint,

D(x+u,y+v,t+δt)−D(x,y,t)=0, (1)

which specifies that the same data is present at time *t* and time t+δt, only shifted in space. To first order, this can be expressed as

$$E_{d}=D_{x}u+D_{y}v+D_{t}\approx 0,$$ (2)

where $E_{d}$ is the error in the data constancy, $D_{x}$ and $D_{y}$ denote spatial derivatives, and $D_{t}$ denotes the temporal derivative at the point $\left(x,y,t\right)$. The second is the spatial smoothness constraint, which specifies that the computed velocity fields contain smooth and continuous motion where possible. This constraint can be expressed as

$$E_{s}^{2}={\left|\nabla u\right|}^{2}+{\left|\nabla v\right|}^{2},$$ (3)

where $E_{s}$ quantifies the overall departure from smoothness, and $\nabla ≐\left(\partial_{x},\partial_{y}\right)$ denotes the gradient operator. The velocity vector field can uniquely be defined by minimizing both these error terms:

$$\underset{u,v}{\min}\left\{\iint \left[\rho \left(E_{d}^{2}\right)+\alpha \rho \left(E_{s}^{2}\right)\right]dxdy\right\},$$ (4)

for some regularization parameter *α* and penalty function ρ. Horn and Shunck used a quadratic penalty, $\rho \left(x^{2}\right)=x^{2}$, but this can lead to inaccuracies if the underlying data contains hard edges and adjacent regions moving in different directions [41]. More accurate velocity vector fields can be obtained by using the Charbonnier penalty, $\rho \left(x^{2}\right)=\sqrt{x^{2}+\beta^{2}}$, for a small positive constant *β* [42]. Eq 4 can be solved by linearizing its corresponding Euler-Lagrange equations, creating a unique velocity vector field *w* (see Solving optical flow equations in Methods and Materials).

### Complex wave pattern detection by critical point analysis

Turbulence studies typically separate activity at different scales based on velocity fields [33]. We similarly implement independent procedures to detect global patterns (plane waves and synchronous activity), which are active across the whole recording area, and complex wave patterns, including source, sink, spiral and saddle patterns, which are characterized by local activity around their central points. Complex spatiotemporal wave patterns, which are analogous to eddies, are organized around critical points where the velocity field has zero magnitude [35]. These complex wave patterns generate distinctive dynamics around their central critical points; in our methodology, we exploit this dynamical property to automatically detect and classify such patterns. In velocity fields, we identify critical points as locations where both *x*- and *y*-components of the velocity are zero by finding intersections of the bilinearly interpolated zero-level contours lines of *u* and *v* [43]. Each critical point is then categorized by the Jacobian matrix,

$$J=\left(\begin{array}{cc} \frac{\partial u}{\partial x} & \frac{\partial u}{\partial y}\\ \frac{\partial v}{\partial x} & \frac{\partial v}{\partial y} \end{array}\right),$$ (5)

which is estimated at the critical point using bilinear interpolation from the corners of the surrounding 4-element cell of recording sites. Depending on the trace (τ) and determinant (Δ) of the Jacobian, critical points are classified as a node (∆>0 and $\tau^{2}>4∆$), focus (∆>0 and $\tau^{2}<4∆$), or saddle (∆<0), and node and focus points are further classified as stable (τ>0) or unstable (τ<0).

**Fig 1:**
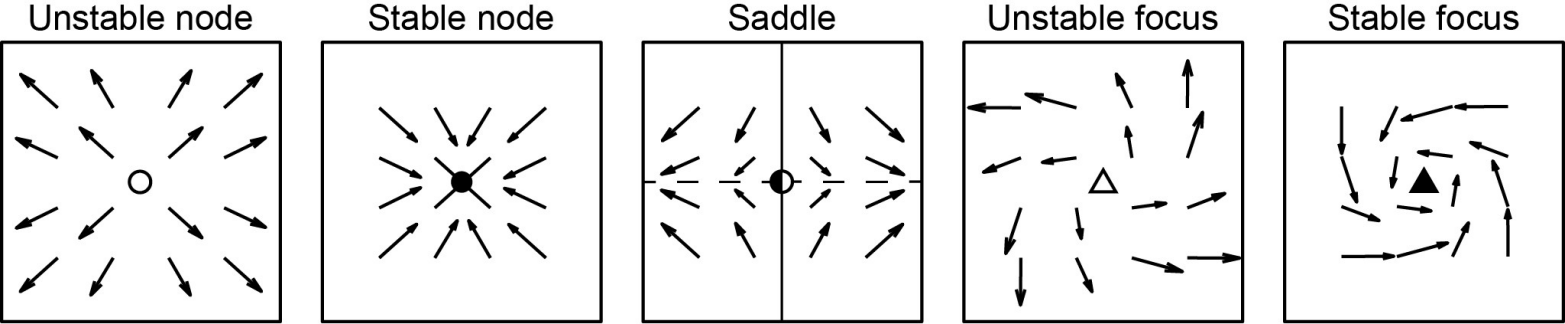
Critical points in vector fields. Schematic of critical point types in velocity vector fields. Dots and triangles show critical points, arrows show typical surrounding vector fields for all 2D critical point classes. Unstable and stable nodes correspond to source and sink patterns respectively, unstable and stable foci correspond to spirals-out and spirals-in respectively. Saddle pattern has stable (solid line) and unstable (dashed line) axes indicated, these axes can occur at any orientation in general.

These classes of critical points correspond to different types of wave patterns (Fig 1): Nodes expand or contract from a critical point, forming sources or sinks, respectively; saddles have one stable axis and one unstable axis, and are typically formed through interactions between different waves; and foci rotate around the critical point, thereby corresponding to spiral waves. In addition to their rotating motion, foci can also involve expansion or contraction from the critical point, forming spiral-out or spiral-in wave patterns, respectively. However, previous studies of spiral waves have not distinguished between spirals-out and spirals-in [12,14,44]. In our methods and toolbox, these patterns can therefore optionally be combined to facilitate direct comparison with other published results.

Although complex wave patterns are classified only by the local properties of their central critical point, they can spread over larger regions of space. We thus develop a method for characterizing the spatial extent of complex wave patterns by using the winding number (Poincaré index), which has a value of +1 for node and focus patterns and -1 for saddle patterns for all closed paths within the pattern’s extent around the location of the critical point [43]. We create approximately circular paths around the location of the critical point, and the winding number is estimated in each of these paths as

$$winding\;number=\frac{1}{2\pi }\overset{\text{n}}{\underset{k=1}{\sum }}\left(\theta_{k+1}-\theta_{k}\right),$$ (6)

where $\theta_{k}$ is the angle of the *k*-th vector around a closed counter-clockwise path with *n* points, angles are subtracted circularly, and where $\theta_{n+1}=\theta_{1}$. We compute the winding number in paths of expanding size around a pattern’s center, and its spatial extent is defined by the largest area within which all computed winding numbers are consistent with the critical point type. This procedure therefore provides an efficient estimate how far wave patterns spread across the cortex, an important property of neural oscillatory activity.

### Synchrony and plane wave detection by order parameters

We next introduce methods for detecting and analyzing simple, large-scale patterns such as synchrony and planar waves by defining order parameters based on the velocity vector fields. We detect planar waves using an order parameter defined as the average normalized velocity [36]:

$$\varphi \left(t\right)=\frac{{||\sum }_{x,y}w\left(x,y,t\right)||}{\sum_{x,y}||w\left(x,y,t\right)||}$$ (7)

This statistic is equivalent to phase gradient directionality [17] except it uses velocity vector fields instead of the phase gradient. Normalized velocity ranges from zero to one, with φ→1 as velocity vectors align to one direction, reflecting coherent motion across the recording area. Plane wave activity is therefore detected at times when *φ* is greater than some threshold value $T_{pw}$, which should be close to one ($T_{pw}=0.85$ by default in the toolbox).

If data has been band-pass filtered to extract the oscillation phase *θ*, we also detect large-spread synchronous activity using another order parameter, which is defined as the resultant vector length of phase across the recording area [45,46],

$$\begin{array}{c} R\left(t\right)=\frac{1}{N}\left|\underset{x,y}{\sum }e^{i\theta \left(x,y,t\right)}\right| \end{array},$$ (8)

where *N* is the number of spatial recording sites in phase maps. The resultant vector also ranges from zero to one, with R→1 as the phase of oscillations at all recording sites align to the same value, reflecting wide-spread synchrony. We note that the order parameter as defined in Eq 8 is similar to that used to characterize global synchrony in coupled phase oscillators [47], and 1-R is commonly defined as the circular variance [45]. Synchronous activity is therefore detected at times when *R* is higher than another threshold value $T_{syn}$, the default of which is $T_{syn}=0.8$ in the toolbox.

### Critical point detection accuracy in simulated data

**Fig 2:**
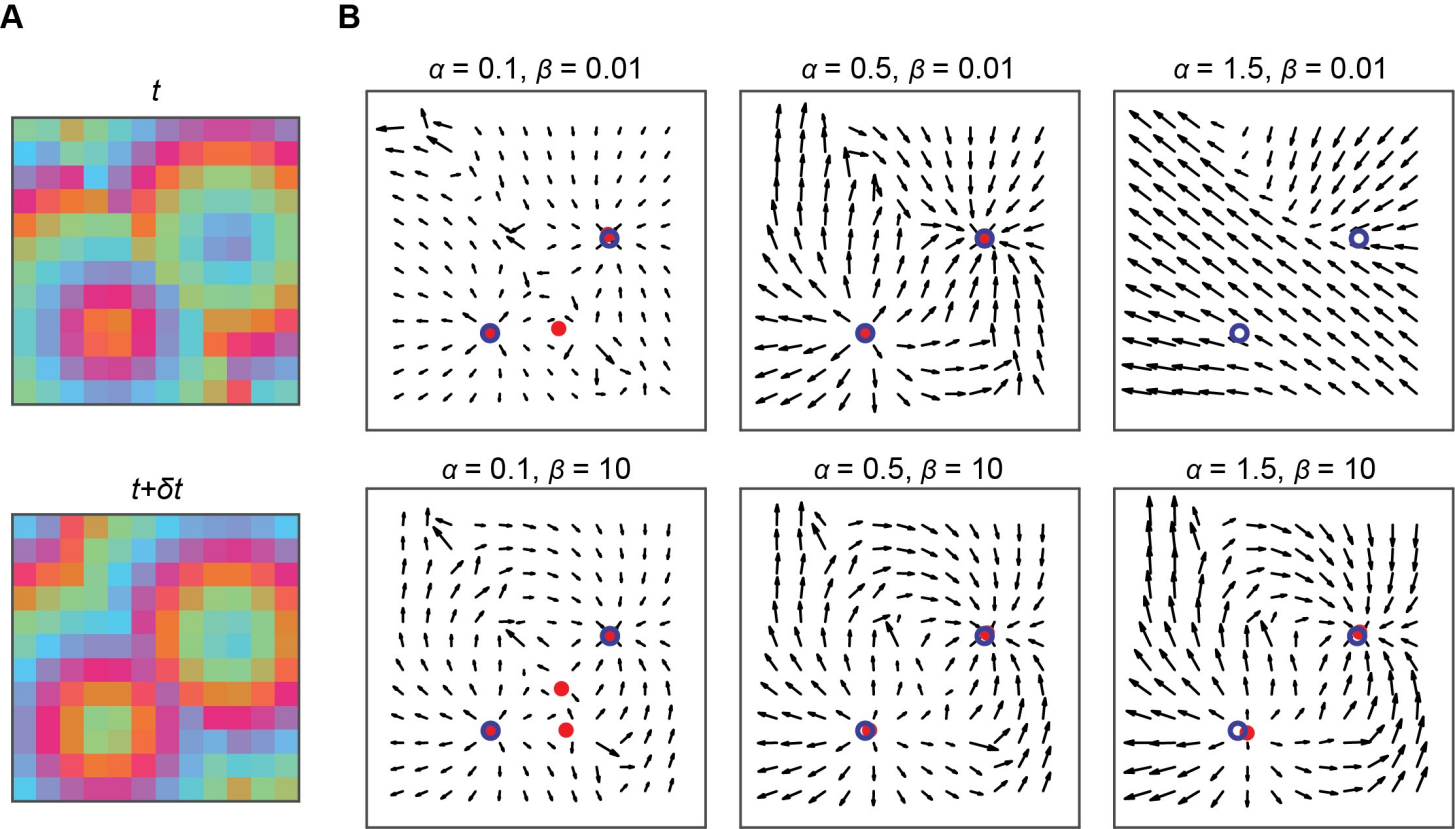
Effect of optical flow parameters on computed velocity fields. **A,** Consecutive snapshots separated by arbitrary time step *δt* of artificial noisy phase data, containing an expanding source and contracting sink pattern, both drifting slowly across the array. **B,** Velocity fields between images in **A** calculated using optical flow with varying smoothness parameter *α* and non-linear penalty parameter *β*. Filled red circles indicate detected critical points, open blue circles indicate true critical point locations.

To test the performance of our pattern detection methods, we generated artificial data sets with simultaneous source and sink patterns active at the same frequency, located at random positions and propagating in random directions within a 12×12 spatial grid (see Simulated data in Methods and Materials). We then added Gaussian white noise, band-pass filtered the signal, calculated velocity vector fields, searched for complex wave patterns in the velocity fields, and compared the detected pattern centers with their true locations. An example of this procedure is shown in Fig 2, which shows calculated velocity fields and pattern centers between two frames of a simulated data set (Fig 2A). The velocity fields depend on two parameters in the optical flow estimation procedure (Eq 4): The smoothness regularization parameter *α*, and non-linear penalty constant *β*.

The smoothness regularization parameter *α* determines the weighting of the smoothness constraint compared to the data constraint, and thereby the overall smoothness of the velocity fields. Small values of *α* generate velocity fields that primarily capture local changes and are therefore sensitive to added noise, potentially leading to the detection of spurious, noise-driven patterns (Fig 2B, left column, α = 0.1). Large values of *α* are more robust to noise, but can over-smooth the data, creating mostly uniform flow fields that do not capture the underlying dynamics (Fig 2B, right column, *α* = 1.5). Reasonable values for *α* can range from ~0.1 to ~20, depending on the size of the data, the dynamics of the propagations, and the level of noise; for example, reducing the spatial sampling frequency of a dataset reduces the number of grid spaces between complex patterns, typically requiring a lower value of *α* to effectively resolve individual patterns. The non-linear penalty constant *β* determines the degree of non-linearity of the penalty functions, with large values β≫1 resulting in a quadratic penalty and small values β≪1 in a more robust non-linear penalty. Small values of *β* give more accurate velocity vector fields for any regions with discontinuous motion in the underlying data [41,48], but we find that such discontinuities are rare in neural recordings, so using large values of *β* generally gives similar results (Fig 2B). In addition, when *β* is large and the equations are effectively quadratic, the optical flow procedure can typically converge much faster.

**Fig 3:**
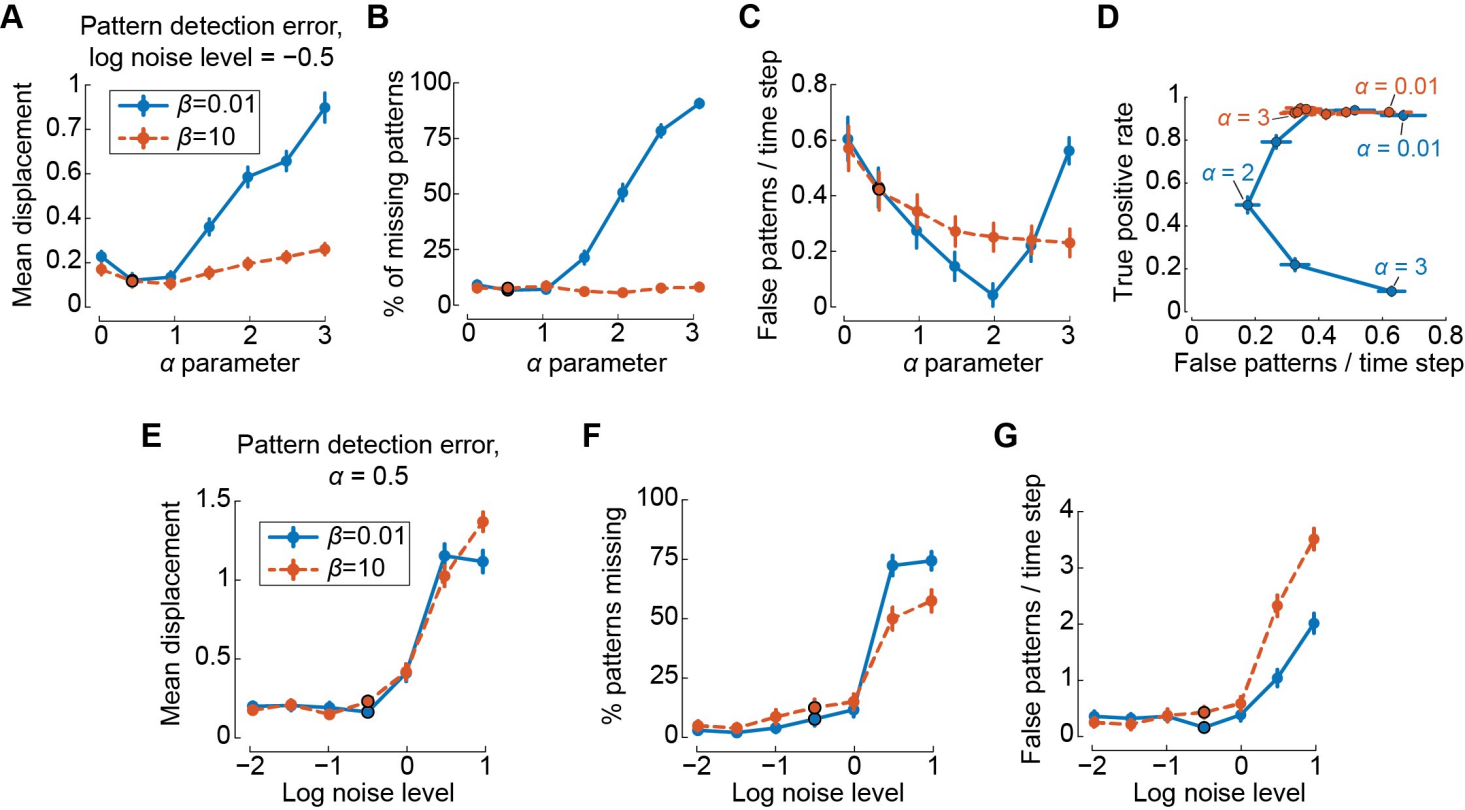
Critical point pattern detection performance in simulated data. **A,** Mean displacement (in grid spaces) between detected and true critical point location across *n* = 50 sequences of simulated random critical point patterns for different values of optical flow parameters *α* and *β*. Error bars indicate SEM. Outlined points indicate *α* parameter value used for plots **D**-**F**. **B,** Mean percentage of true patterns missed or misclassified by pattern detection algorithm. **C,** Mean number of extra, spurious patterns detected per time step. **D,** Mean false patterns detected per time step (false positive rate) against the percentage of patterns correctly identified (true positive rate) for different parameter values. **E-G,** Same as **A**-**C**, but showing performance under different levels of noise with a fixed value of *α*. Noise levels are given as the common logarithm of the standard deviation of added white noise relative to average signal amplitude. Outlined points indicate noise level used for plots **A-C**.

The choice of appropriate values for *α* and *β* cannot be fully automated for a real dataset without making assumptions about the dynamics of the data. However, pattern detection accuracy can be evaluated in simulated datasets with specified properties and pattern dynamics, which can then be used to guide parameter choices in real data. Fig 3 illustrates the effectiveness of the pattern detection algorithm for one such set of properties and dynamics (see Simulated data in Methods and Materials). The detected spatial position of patterns is most accurate when using small values of α (α≤1, Fig 3A). Using a quadratic penalty function (β=10) generally gives more consistent results across a range of α values than a non-linear penalty function (β=0.01) and results in fewer missing patterns (Fig 3B), but using the non-linear penalty can give a lower false positive rate (Fig 3C). Plotting the true positive rate against the false positive rate provides a clear way to examine the effectiveness of pattern detection across a range of parameters (Fig 3D). We generally recommend using large values of *β* when examining new data sets, as this provides more reliable performance and faster processing overall. Additionally, pattern detection is largely unaffected by noise if the variance of the noise is equal or less than the variance of the pattern oscillations and remains fairly accurate for significantly greater noise levels (Fig 3E–3G).

### Propagating wave patterns in large-scale neural data

**Fig 4:**
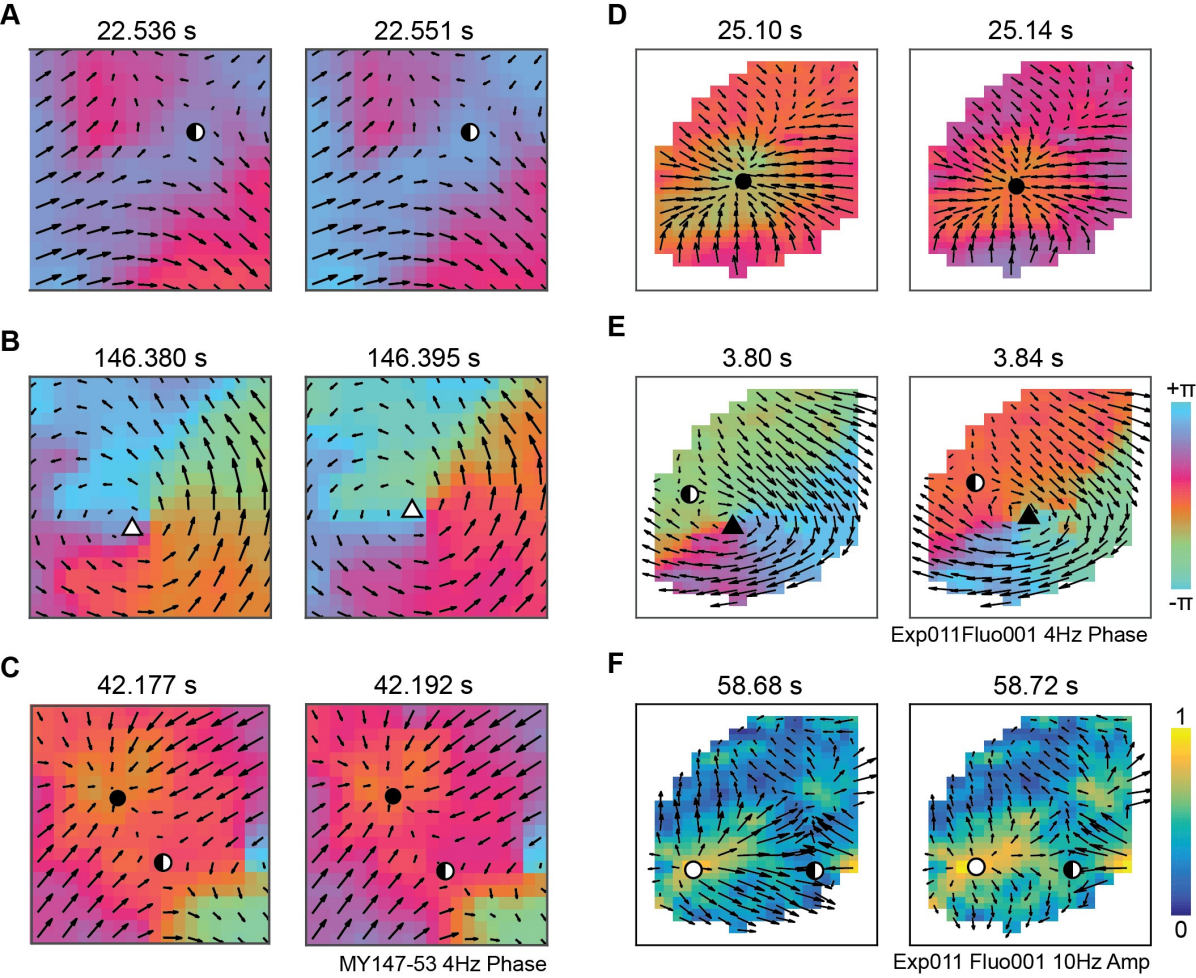
Complex wave patterns in marmoset area MT and mouse cortex. **A, B, C,** Complex wave patterns in phase maps of 4 Hz LFP oscillations during ongoing activity recorded from marmoset area MT. Velocity vector fields (black arrows) were calculated between consecutive phase maps separated by 1 ms; we use larger time gaps between snapshots here to show wave propagation more clearly. Critical points are indicated by symbols corresponding to the classes in Fig 1. **D, E,** Complex wave patterns in phase maps of 4 Hz optical voltage imaging oscillations in awake mouse cortex. Velocity vector fields are calculated from phase maps separated by 20 ms. **F,** Localized propagating activity in amplitude maps of 10 Hz optical voltage imaging oscillations in awake mouse cortex.

To validate our methods and test for wave pattern activity in real neural data from different scales and imaging techniques, we examined previously published LFP recordings from the MT area of anaesthetized marmosets [49] and optogenetic voltage imaging recordings from a complete cortical hemisphere in awake mice [50] (see Experimental recordings in Methods and Materials). Using our methodology, we searched for wave patterns within the phase and amplitude of oscillations across a range of frequency bands. Both datasets exhibited a rich repertoire of wave patterns which were successfully detected. Some examples of common pattern activity for each modality are shown in Fig 4.

In delta-band phase of the marmoset data, complex waves were commonly present across the whole 16 mm^2^ recording area, including saddle (Fig 4A) and spiral-out (Fig 4B) patterns. We also observed multiple complex wave patterns active simultaneously in different areas of the cortex, as shown for sink and saddle patterns in Fig 4C. In the mouse data, complex waves were present in the phase of slow (4 Hz) oscillations, and these waves sometimes spread across the whole cortical hemisphere, including sink (Fig 4D) and spiral-in (Fig 4E) patterns. Large-scale propagating patterns were also present in the amplitude of 10 Hz oscillations, where multiple spreading patterns often interacted to form saddles (Fig 4F). These examples demonstrate that complex wave patterns are present at multiple scales of brain activity, and that these patterns can be detected and quantified through our methodology.

### Analysis of wave pattern properties

**Fig 5:**
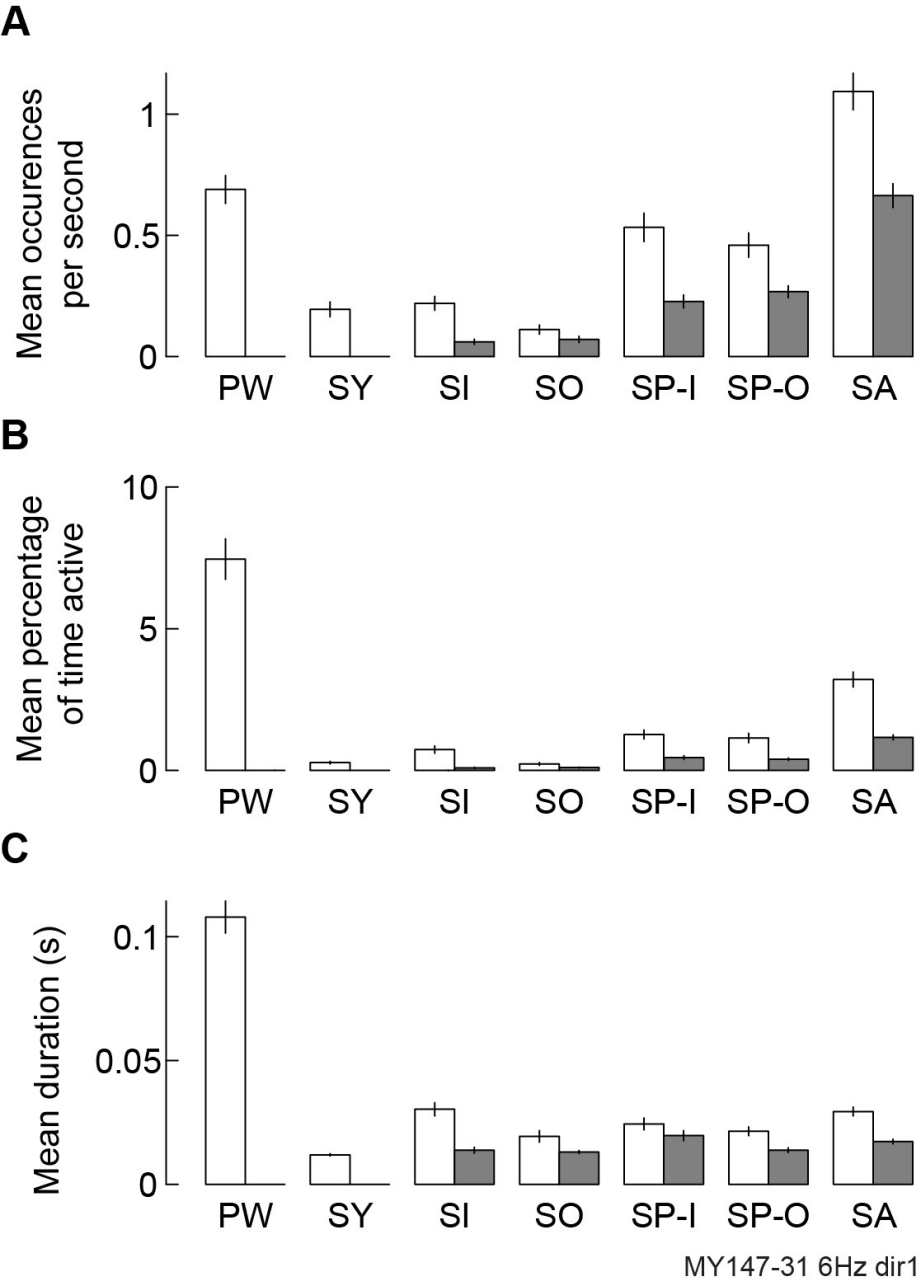
Wave pattern properties in real data compared to noise-driven surrogate data. **A,** Mean number of 6 Hz phase patterns detected per second per trial in real data (white bars, marmoset LFPs during dot-field stimulus) and surrogate data (shaded bars, white noise with equal mean and variance per recording channel to real data). Error bars indicate SEM across 100 trials for both datasets. PW, plane wave; SY, synchrony; SI, sink; SO, source; SP-I, spiral-in, SP-O, spiral-out, SA, saddle. **B**, Same as **A**, but showing percentage of recording time active. **C,** Same as **A**, but showing mean pattern duration.

Having presented our pattern detection methods, we now demonstrate how these techniques can be used to examine the properties and dynamics of waves patterns in more detail, and how these properties can be further related to brain function. Directly tracking simple and complex wave patterns allows their location, movement direction, prevalence, duration and other properties to be collated across many occurrences. To validate the results of the pattern detection procedure, the properties of patterns detected in a real dataset can be compared to those of patterns detected in a surrogate dataset comprised of noise with similar characteristics to the original data (see Pattern detection parameters and result validation in Methods and Materials). The processes of band-pass filtering and velocity vector field estimation can smooth data and may therefore generate spurious wave patterns in noise-driven surrogates. However, these patterns in surrogate data are typically more localized and transient than real neural wave patterns and can therefore be mostly removed if the minimum pattern spatial extent and duration parameters are sufficiently large. In neural recordings with genuine wave pattern activity, all pattern types will typically occur more frequently (Fig 5A), be present for a larger proportion of recording time (Fig 5B) and last longer per occurrence (Fig 5C) than equivalent patterns in noise-driven surrogates.

**Fig 6:**
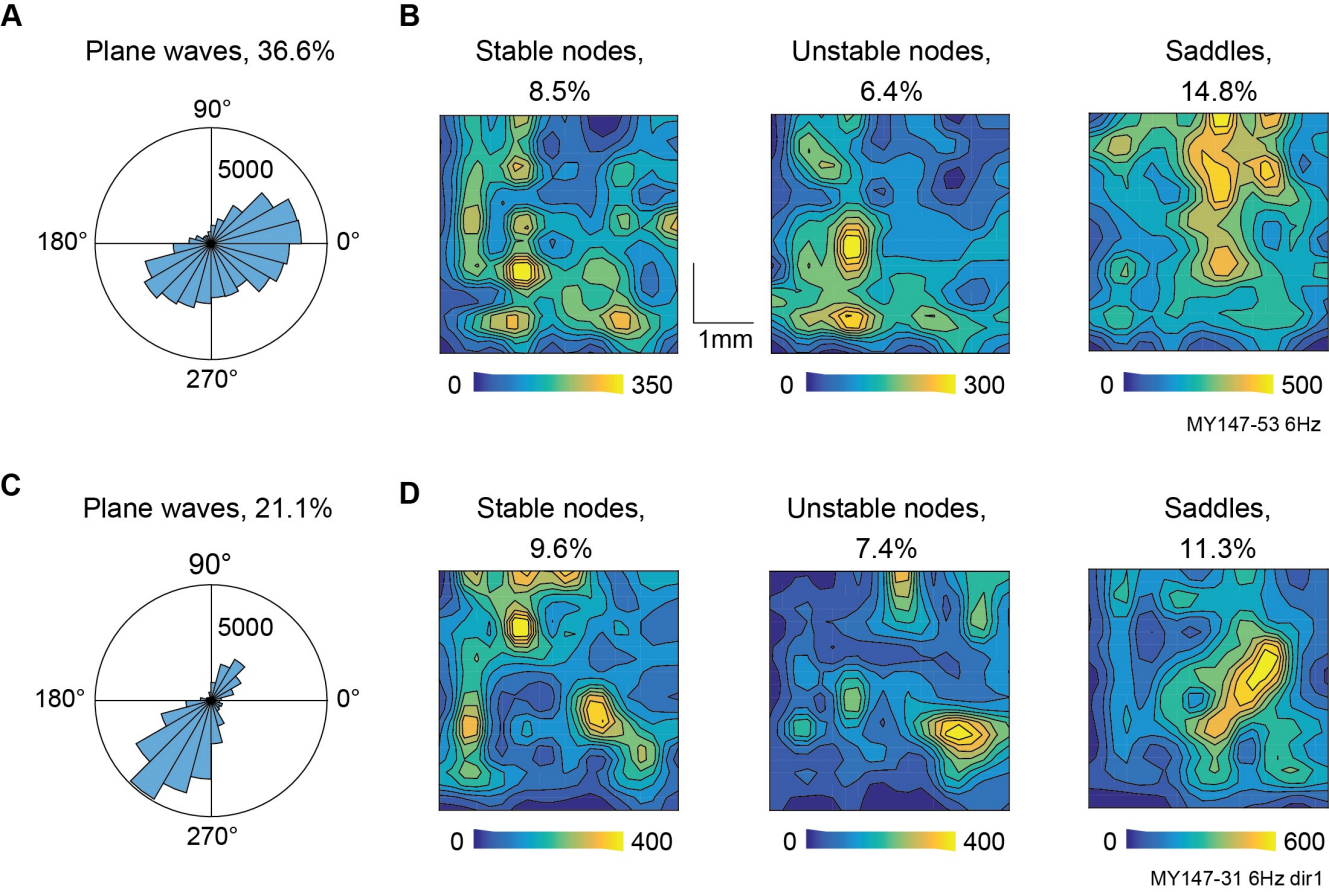
Distributions of plane wave propagation direction and complex pattern center location in ongoing and stimulus-evoked PVFs. **A,** Histogram of plane wave propagation direction in 6 Hz phase of ongoing activity. Also shown is the percentage of time across the recording in which plane wave activity occurred. **B,** Spatial distribution within the recording array of critical points across all occurrences of node and saddle patterns. Note that foci have been combined with nodes for this analysis. **C, D,** Same as **A**, **B**, but for a recording visually evoked by moving dot fields.

The properties of wave patterns can vary depending on brain state, recording location, or cognitive task, revealing relevant dynamical changes in the recorded neural system. An example of this is shown in Fig 6, which compares properties of patterns in spontaneous and stimulus-evoked phase velocity fields from the same animal. During ongoing activity (sustained blank screen stimulus), plane waves were active for much of the recording time and propagated in a range of directions (Fig 6A, mean resultant vector length 0.28). Complex wave patterns were also common and did not form randomly in space. Instead their central critical points were clustered around preferred locations (Fig 6B), which were situated at different points in the recording array for node and saddle patterns. When relevant stimulus was presented (coherently propagating dot fields turned on and off every two seconds), relatively fewer plane waves were active overall, but their propagations were more tightly distributed around one preferred direction (Fig 6C, mean resultant vector length 0.42). The presence of stimulus also affected the overall prevalence of critical point patterns, increasing the number of stable and unstable nodes and decreasing the number of saddles, and changed their patterns of distribution across space (Fig 6D). Our methods can therefore be used to quantify changes in spatiotemporal pattern dynamics driven by different stimuli, cognitive tasks or behavioral states.

**Fig 7:**
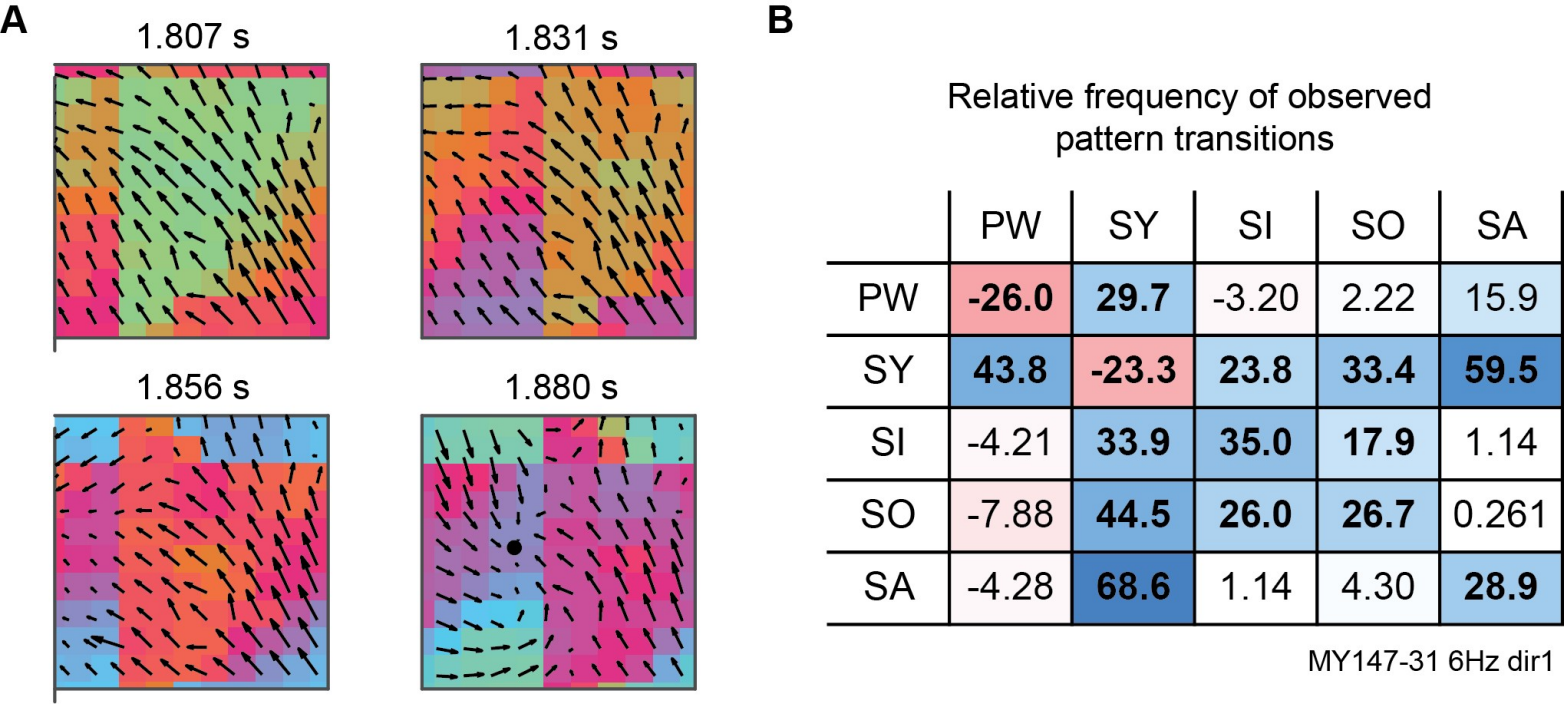
Tracking pattern evolution dynamics. **A,** Snapshots of 6 Hz phase and phase velocity fields from marmoset area MT LFPs during dot field stimulus, showing evolution from a plane wave pattern to a sink pattern. Black dot marks stable node location. **B,** Mean percentage difference between number of observed and expected pattern transitions (within 50 ms) for recording shown in **A**. Rows give initial pattern, columns give following pattern. Bold values indicate significant differences between observed and expected counts across all trials (p<0.05, paired t-test with Bonferroni correction). PW, plane wave; SY, synchrony; SI, sink; SO, source; SA, saddle. Node and focus critical points are combined.

Detected wave patterns can also be processed to reveal their temporal evolution dynamics. Brain activity evolves between different activity patterns in a complex and non-random way, but the mechanisms of these transitions are not well-understood [13,15]. Our methodology provides an ideal framework for exploring such dynamics: Once all patterns in a recording have been identified, common pattern transitions and motifs can easily be identified. We demonstrate some of these evolution dynamics in stimulus-evoked LFP recordings (see Pattern evolution dynamics in Methods and Materials). Patterns were typically active for tens to hundreds of milliseconds, often then transitioning into a different pattern (Fig 7A). The total number of transitions between all pairs of pattern types were counted across a recording, and the significance of these observed counts was established by comparison to the expected number of counts if all patterns began and ended at random times (Fig 7B). Using this simple analysis, we observed that periods of plane wave and synchronous activity were usually interspersed by other pattern types, synchronous activity was highly likely to transition to or from all other pattern types, and patterns commonly evolved from sources to sinks and vice versa. This analysis illuminates the temporal dynamics of the spatiotemporal activity patterns present in the recording and provides quantitative measurements which can be linked to cognitive tasks or used to constrain models of cortical dynamics. Similar analyses can also facilitate tracking the movement of neural structures of interest across brain regions [15], detecting repeated motifs in pattern dynamics [13], or examining gradual changes in pattern dynamics corresponding to changes in brain states [50].

### Phase and amplitude patterns

**Fig 8:**
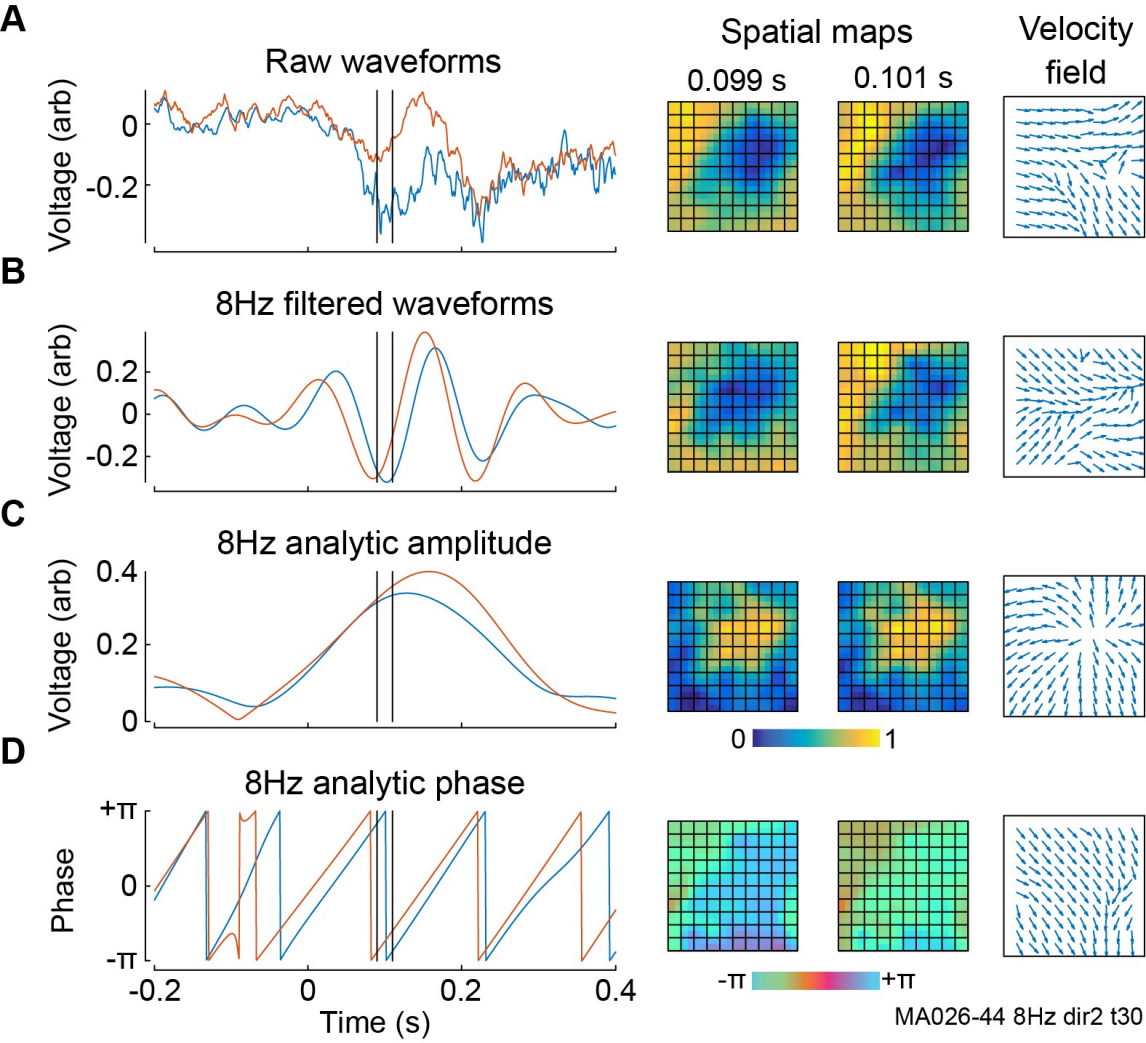
Oscillatory filtering and velocity vector field calculation in LFPs from marmoset visual area MT during dot field stimulus. **A,** Left: LFP waveforms from two nearby recording sites. Black lines indicate times at which snapshots are shown. Stimulus onset was at 0 s. Centre: Snapshots of LFPs across all recording channels at the indicated times. Right: Velocity vector field estimating the motion between the two snapshots. **B,** Same as **A**, but with waveforms filtered with Morlet wavelets to extract 8 Hz oscillations. **C,** Same as **B**, but only the amplitude of the 8 Hz oscillations. **D,** Same as **B**, but only the phase of the 8 Hz oscillations. All velocity fields have normalized average magnitude.

In neural recordings, amplitude and phase data at the same frequency reflect different properties of brain activity, with amplitude representing a combination of the coherence and overall activity of a local ensemble and phase representing the timing of its oscillations. Accordingly, these signals typically contain different spatiotemporal patterns, and both phase and amplitude patterns can be relevant and informative. Fig 8 illustrates the spatiotemporal profile of raw LFP data, filtered LFP data, and the amplitude and phase of filtered LFP data, again taken from marmoset visual area MT. The spatiotemporal dynamics in the raw data (Fig 8A) primarily reflect those in the oscillations with greatest power, but also contain a large amount of noise from other frequencies. Filtering the data to a narrow-band signal (Fig 8B) reduces the noise by extracting the patterns present in the chosen frequency band alone, but these wave patterns are typically complicated as they are influenced by two different types of propagating activity: amplitude patterns (Fig 8C), which capture the movement of the overall shape of the wave and travel at the group velocity [51], and phase patterns (Fig 8D), which capture the progression of timing differences between electrodes and travel at the phase velocity.

In a general oscillating system, the phase and amplitude are independent properties which have no a priory reason to affect each other. However, there is some evidence that phase and amplitude patterns can be related in some neural systems: Phase patterns in rabbit sensory cortices are more commonly observed around the formation of new amplitude patterns [52], and spiral waves in mammalian neocortex exhibit consistently reduced amplitudes at their centers [12,44]. Examining both phase and amplitude separately may therefore uncover similar relationships in other experimental protocols and can reveal a more comprehensive understanding of the underlying dynamics of cortical circuits. For example, two simple patterns can be resolved from the complicated activity in Fig 8B: A gradually expanding activation from a point near the center of the recording array, as revealed by the amplitude velocity field in Fig 8C, and a plane wave propagating across the recording area, as revealed by the phase velocity field in Fig 8D.

### Analysis of spatiotemporal modes

Whilst direct identification of wave patterns in velocity fields as described in the previous sections allows for patterns’ individual dynamics to be fully characterized, the procedure does not specify the extent to which these patterns contribute to the overall spatiotemporal dynamics of a recording. To address this, we introduce a complementary method for studying wave activity in neural recordings by using velocity field decomposition, which finds low-dimensional spatiotemporal modes that capture the majority of variance in the system. Dimensionality reduction techniques are commonly used for uncovering underlying neural mechanisms of brain function [6]. However, the majority of existing techniques use principal component analysis (PCA) or similar procedures that decompose data into independent spatial and temporal modes, obscuring activity that is not time-space separable such as propagating waves and patterns [53]. Some studies have used decomposition techniques to specifically detect waves by examining phase gradients in complex decompositions of data [26,29]. To identify dominant spatiotemporal patterns in our framework, we again obtain inspiration from the field of turbulence, in which dimensionality reduction is often directly applied to velocity fields, capturing low-dimensional spatiotemporal dynamics [33].

In turbulence, dimensionality reduction can be performed through a variety of different decomposition methods, including Reynolds decomposition, principal component analysis (or proper orthogonal decomposition), and dynamic mode decomposition [54]. These techniques find modes capturing the majority of the energy in the system, which is not well-defined for velocity fields of neural data as it is in fluid flows, but some of these methods nonetheless can be adapted to find low-dimensional representations of the primary spatiotemporal dynamics of a neural recording. We implement a simple singular value decomposition (SVD) to extract the dominant spatiotemporal patterns from a time series of velocity fields in an efficient and parameter-free way.

To reorganize the velocity fields $\left(u\left(x,y,t\right),v\left(x,y,t\right)\right)$ into standard form for decomposition with variables in columns and observations in rows, we combine spatial dimensions and rearrange indices to define $\left(\tilde{u}\left(t,r\right),\tilde{v}\left(t,r\right)\right)$, for time *t* and recording site *r*. We then use two alternate approaches to combine *ũ* and *ṽ*. In the first approach, we concatenate the two matrices across recording sites to define the real matrix ${\tilde{w}}_{re}\left(t,r'\right)=\left[\tilde{u}|\tilde{v}\right]$. In the second, we represent the velocity field as a complex number to form the complex matrix ${\tilde{w}}_{co}\left(t,r\right)=\tilde{u}\left(t,r\right)+i\tilde{v}\left(t,r\right)$. In either case, the singular vector decomposition (SVD) is defined as

$$\tilde{w}=T\Sigma R^{*},$$ (9)

where $\tilde{w}$ denotes **${\tilde{w}}_{re}$** or ${\tilde{w}}_{co}$, ***T*** and ***R*** are unitary matrices, * denotes the conjugate transpose, and *Σ* is a rectangular diagonal matrix of positive numbers $\sigma_{i}$, called the singular values [55]. This operation finds orthogonal linear combinations of recording sites that explain the greatest variance in the velocity fields, and is closely related to PCA: if $\tilde{w}$ has been shifted so that each recording site has zero sample mean, then ***R*** comprises exactly the principal component loadings, and $\sigma_{i}^{2}$ are the principal component scores [56]. However, normalizing the velocities at each recording site is counterproductive in this application, as biases in propagation direction are an important component of wave dynamics. The *k*-th spatial mode, defined by the velocity field in the *k*-th column of ***R***, explains a proportion of the overall variance given by $\sigma_{k}^{2}/\sum_{i}\sigma_{i}^{2}$, and has a time course given by the *k*-th row of ***T***.

**Fig 9:**
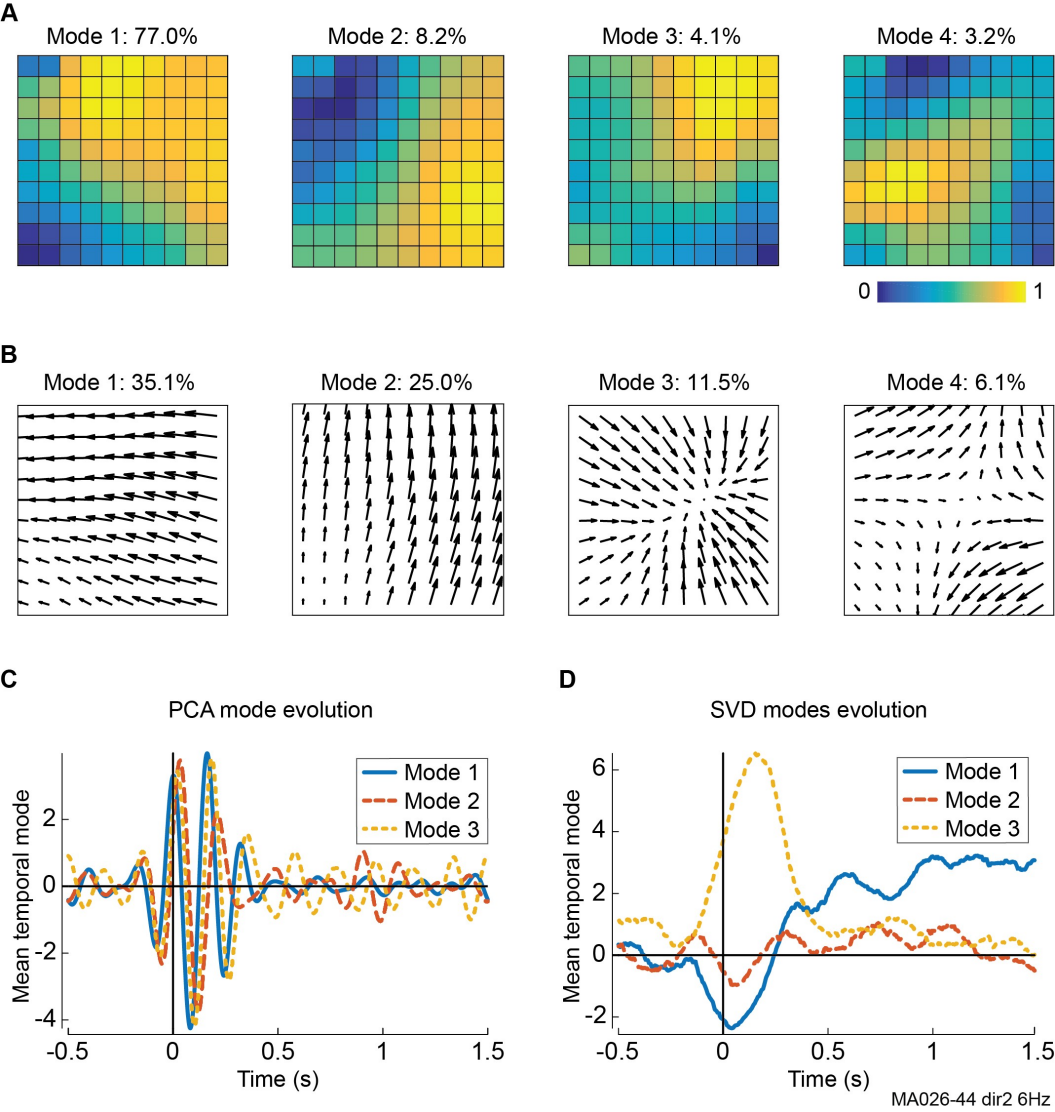
Dominant principal component analysis and vector singular value decomposition modes in 10Hz marmoset LFP oscillations during dot-field stimuli. **A,** Top spatial PCA modes of filtered LFPs, with percentage variance explained. **B,** Top spatial SVD modes of phase velocity fields, showing coherent spatiotemporal activity patterns. **C,** Temporal evolution of PCA modes, averaged across all trials. Stimulus onset at 0 s. Non-causal effects are due to time smoothing of signal filtering. **D,** Same as **C**, but for SVD modes.

The vector SVD procedure is closely related to traditional PCA methods, as both techniques reduce the dimensionality of a dataset by extracting patterns that comprise the bulk of the variance and their evolution over time. The differences between these approaches are illustrated in Fig 9, which again shows marmoset LFP data during moving dot-field stimulation. PCA typically decomposes data into orthogonal spatial modes (Fig 9A), which comprise linear combinations of recording sites [6]. Vector SVD instead processes the velocity vector fields to extract spatial modes which are vector fields themselves (Fig 9B), and therefore represent distinct propagation patterns in the underlying data. In both cases, each spatial mode has a corresponding time-course (or temporal mode), describing its evolution across the duration of a recording (Fig 9C and 9D).

Although the dominant PCA modes explain more variance than their SVD counterparts, their temporal components reveal structured interactions between the dominant spatial modes (Fig 9C), generating spatiotemporal activity patterns which are difficult or impossible to determine directly from the PCA modes. In contrast, SVD spatial modes directly reflect these spatiotemporal patterns, and their evolution over time represents the strength of different pattern types in response to stimulus. In Fig 9, stimulus onset generates large, clear changes in spatiotemporal pattern dynamics revealed by SVD (Fig 9D): Sink pattern activity increases dramatically but transiently (shown by the large deflection in mode 3); plane waves (modes 1 and 2) increase in activity more modestly, but change direction soon after stimulus onset (as indicated by the sign change of mode one at 300 ms) and are sustained for a longer period. These results suggest that stimuli can directly affect the dynamics of propagating wave patterns, but that these changes are obscured when using PCA or other decomposition methods that separate space and time.

**Fig 10:**
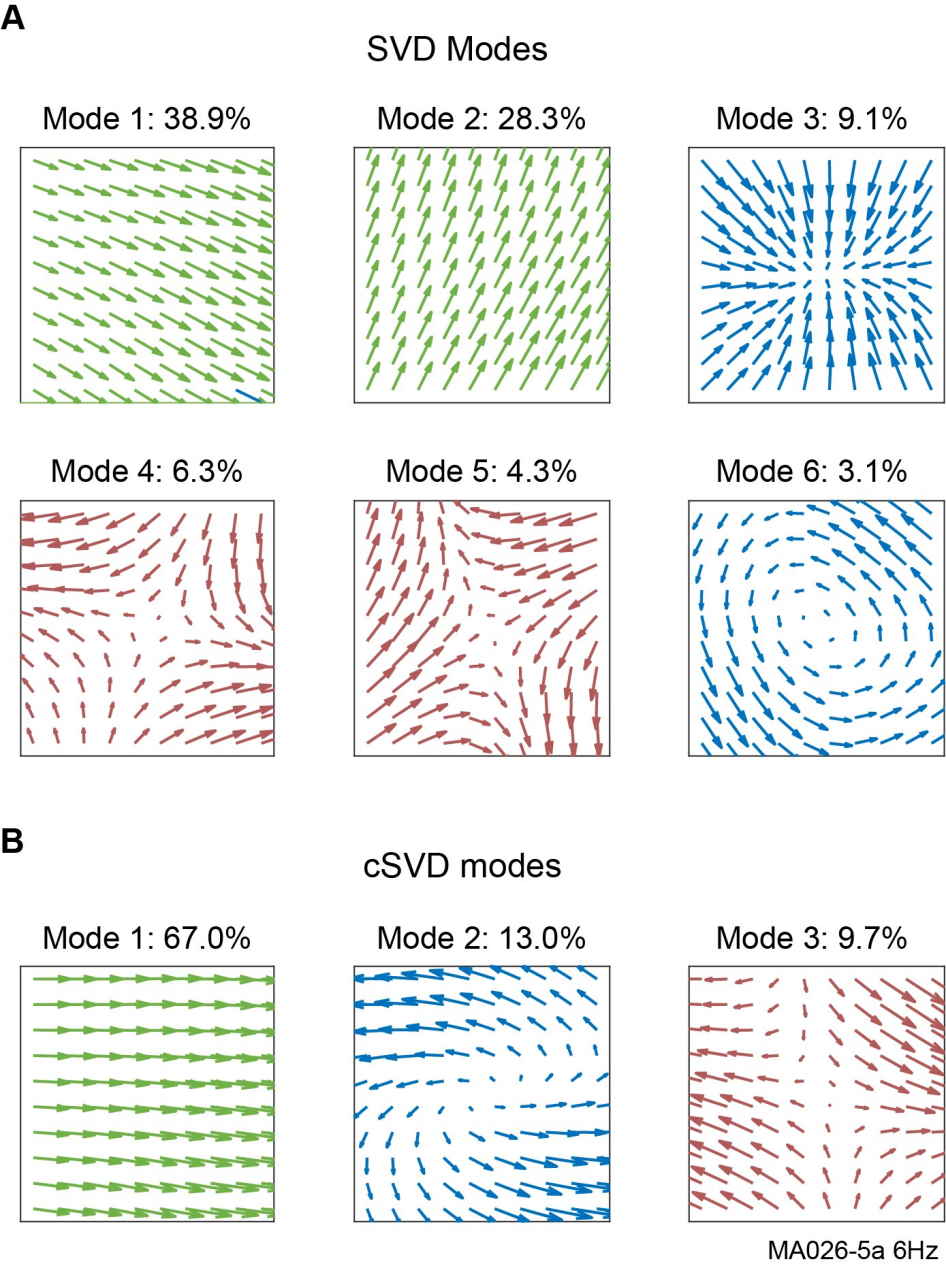
Real and complex vector singular value decomposition modes in 10 Hz phase velocity fields. LFPs were taken from the same recording as in Fig 9, but during ongoing activity. **A,** Top spatial SVD modes in velocity fields, with percentage variance explained. Modes correspond to spatiotemporal patterns, which are scaled but not rotated as they evolve over time. **B,** Top spatial cSVD modes, with percentage variance explained. These modes are both scaled and rotated over time, effectively combining multiple SVD modes as indicated by colors: cSVD mode 1 contains SVD modes 1 and 2; cSVD mode 2 contains SVD modes 3 and 6; and cSVD mode 3 contains SVD modes 4 and 5.

We find that in both spontaneous and stimulus-evoked LFP recordings, velocity vector fields in phase and amplitude at all frequencies display consistent dynamics: the most dominant modes typically reflect orthogonal directions of plane wave motion, and the next most dominant modes contain complex patterns including sources, sinks, spirals and saddles (Fig 10A). Despite these similarities, the disparities between spatial modes in different recordings or conditions can reveal major differences in the underlying pattern dynamics, including the primary directions of plane wave motion, the center location of complex patterns, and the relative prevalence of different pattern types. As an example, we compare the dominant SVD modes during stimulus-evoked activity in Fig 9B to those during ongoing activity in the same recording (Fig 10A). The four most dominant modes represent the same activity patterns, but they display slightly different dynamics: The primary propagation direction of plane waves changes (shown by the direction of mode 1); the central locus of source, sink and saddle activity changes location (shown by the critical point in nodes 3 and 4); and ongoing activity overall contains more plane wave and less source, sink and saddle activity (as revealed by the percentage of variance explained).

### Real and complex singular value decomposition

In the SVD method discussed thus far, each class of spatiotemporal pattern may be represented across multiple modes (e.g., modes one and two both reflect plane wave activity), making their overall prevalence more difficult to calculate. To address this issue, we also implement a modified SVD procedure that we call complex singular value decomposition (cSVD), which treats velocity vectors as complex numbers. In this approach, temporal modes have both a real and imaginary component, allowing spatial modes to both scale and rotate over time: The amplitude of the temporal mode gives the relative strength of the pattern, and the argument of the real and imaginary components gives the angle by which all vectors are rotated. This approach effectively combines real SVD modes together (Fig 10): Modes with plane waves travelling in any direction are combined, as are modes containing source, sink and spiral patterns with the same center location, or saddles with the same center location. This allows the overall relative contribution of each type of activity pattern (plane waves, expanding or contracting waves, saddle patterns) to be accurately evaluated, but information about the direction of patterns is removed to the complex time evolution.

## Discussion

In this paper, we have introduced a methodological framework and associated MATLAB toolbox for the classification and analysis of propagating wave patterns in neural recordings, and illustrated these methods using simulated data, LFP recordings from marmoset visual area MT and whole-brain optical imaging data from mouse cortex. The toolbox is freely available under an open source agreement from [https://github.com/BrainDynamicsUSYD/NeuroPattToolbox]. As we have demonstrated, our methods provide a framework for uncovering the spatiotemporal organization principles of these patterns and for examining how they are related to brain function.

We have introduced velocity vector fields to characterize how neural oscillatory signals change across space and time. Based on these vector fields a range of mathematical techniques including order parameters, critical point analysis and winding number calculation are uniquely combined to detect a diverse range of wave patterns and to characterize their key spatiotemporal organization properties. Our methods thus build upon the application of optical flow analysis for detecting wave patterns developed in previous studies [15,57,30,58]. As we have demonstrated, order parameters can be used to detect the presence of large-scale plane waves or synchronous activity, and critical point analysis can detect complex wave patterns, comprising sources, sinks, spirals-in, spirals-out, and saddles. Calculation of the winding number around critical points can then be used to measure the precise size of wave patterns, which may be useful in future studies to examine the spatial scale of neural features and effects across different frequencies [59]. These approaches allow multiple classes of waves to be tracked simultaneously and systematically.

Applying these methods to experimental data, we successfully identified both small-scale wave patterns in LFP recordings from anaesthetized marmoset visual cortex and large-scale patterns in whole-brain optical recordings from awake mice. In both datasets, multiple coexisting patterns were commonly active and all patterns were significantly more prevalent than in noise-driven surrogate data (Fig 5). Furthermore, we showed that visual stimulation can change the direction of plane wave activity and the position of complex waves in marmoset area MT (Fig 6), and that these waves evolve between different types in a structured way beyond what is expected by chance (Fig 7). These results are consistent with previous studies associating visual stimuli and traveling waves [9,10,19], and showing that neural dynamics evolve following preferred pathways [13,15,60]. However, unlike previous work, our methodology allows these patterns and their dynamics to be simultaneously detected and quantified, and places them into a framework of explicit pattern behavior to more precisely study underlying neural dynamics.

In our methods, dominant spatiotemporal activity patterns can be extracted from a recording using novel vector field decomposition methods. These present a promising approach to the task of dimensionality reduction in large-scale neural recordings. Current dimensionality reduction methods typically process data into separable temporal and spatial modes which reproduce the dynamics of a recording [6]. However, these approaches find population structures that are often dominated by single-cell response properties and correlated activity [61], and do not adequately capture activity patterns that are not time-space separable, such as propagating waves [53]. In contrast, vector field decomposition specifically targets propagating waves by directly extracting spatiotemporal pattern modes from data. We showed that stimulus onset in marmoset LFP recordings generated complicated responses in spatial PCA modes that are difficult to interpret, but clear effects on the activity of spatiotemporal pattern modes. We also showed that the dominant spatiotemporal modes are consistent across recordings but change in dynamics depending on cognitive function. In the future, this approach could be useful for examining how sensory stimuli and cognitive tasks affect wave dynamics of population-level responses, and for visualization and exploration of the underlying spatiotemporal activity in large neural data sets. Together, the detailed wave pattern tracking approach, and broad, parameter-free velocity decomposition approach provide a comprehensive analysis of spatiotemporal activity patterns in neural recordings.

There are many ways that our methodology can be extended to explore spatiotemporal neural pattern dynamics beyond what has been presented in this paper. For instance, plane waves and synchronous activity are currently detected as global patterns active across the whole recording area, but it would be advantageous (particularly in large-scale recordings) to identify discrete local regions exhibiting these patterns. This would support accurate simultaneous analysis of brain areas displaying different dynamics, be useful for studying the spread of synchrony or plane wave propagations, and provide consistency with the localized nature of complex patterns as defined by the winding number. However, implementing localized order parameters across regions of different sizes would significantly slow the pattern detection procedure and introduce additional free parameters to the framework. Future work may develop more efficient methods to characterize localized regions displaying synchrony or planar propagations, potentially allowing entire cortical sheets to be fully and dynamically segmented into multiple interacting patterns.

Additionally, our methods can be further extended to explore currently unknown mechanisms of wave pattern interactions in the brain. Firstly, localized patterns with complex dynamics that are active simultaneously may directly interact. Such interactions are prevalent in modelling studies including spiking neural networks [37] and neural firing rate models [62], and they are theorized to be directly involved in distributed dynamic computation [25]. In experimental studies, interactions between sharp-wave ripple patterns in rat hippocampus can result in their reflection or annihilation [63], but more complex interactions have not been examined. Secondly, patterns may interact across oscillations at different frequencies. Currently, the phase of low-frequency oscillations is known to influence the amplitude of high frequency oscillations in the brain [64,65], but it is not clear how this cross-frequency coupling actually influences or is influenced by the underlying patterns in these systems. Finally, wave pattern dynamics may interact across multiple spatial and temporal scales in more complex ways, creating cascades of pattern dynamics comparable to energy cascades in turbulence studies [66]. By effectively detecting and analyzing neural spatiotemporal activity patterns simultaneously across multiple scales, our methods provide a framework for further exploring these key questions in future studies.

## Methods and materials

In this section we describe how the methodological framework introduced in the Results section is implemented in the NeuroPatt toolbox and outline how user-set parameters affect computations. We also briefly describe the experimental protocols of the data shown in the figures of this manuscript. The NeuroPatt Toolbox was written in MATLAB 2016b, and is freely available from [https://github.com/BrainDynamicsUSYD/NeuroPattToolbox].

**Fig 11:**
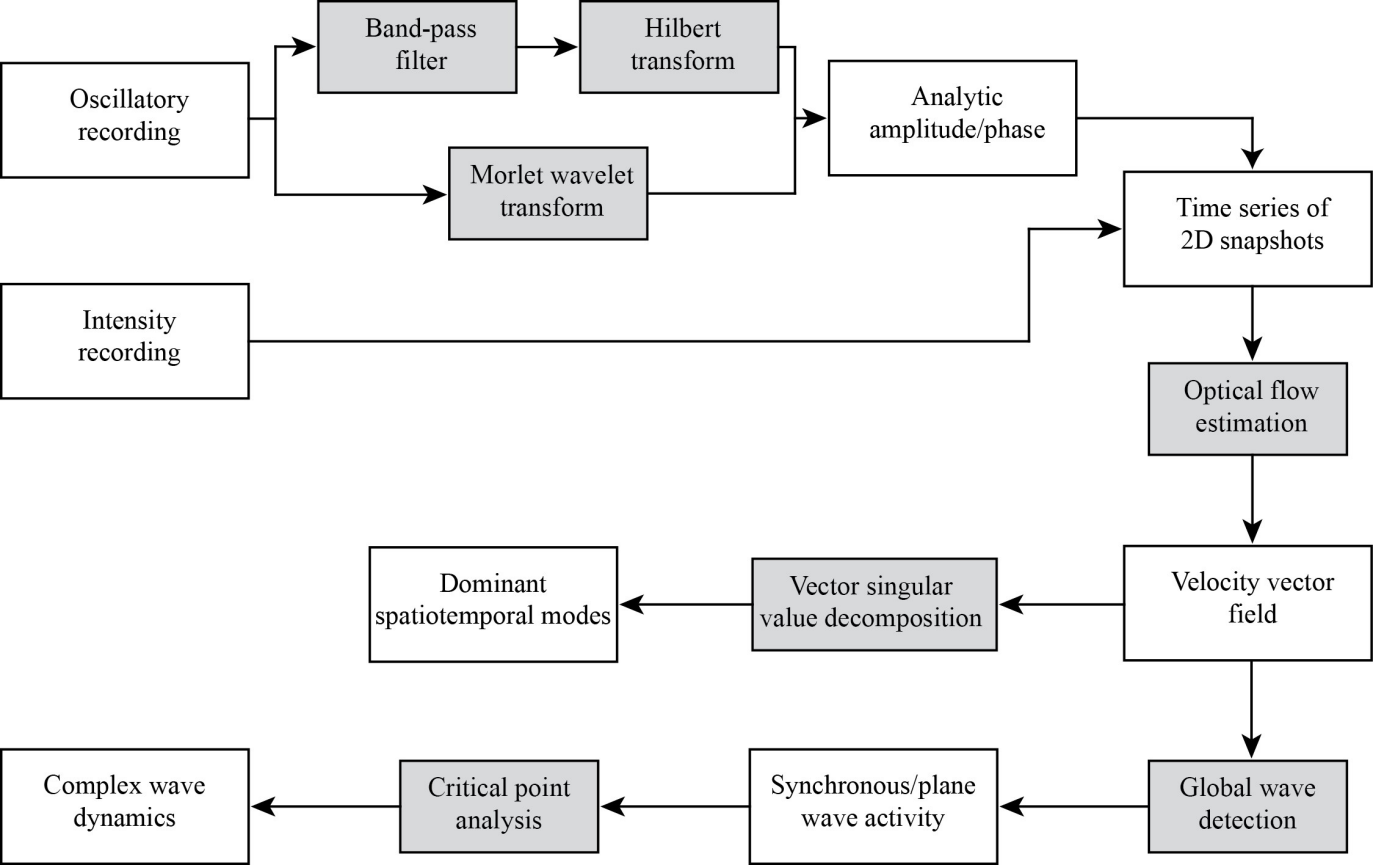
Schematic of data flow in the methodological framework and NeuroPatt toolbox. Neural recordings can either contain oscillatory (e.g. EEG, LFP) or intensity (e.g. VSD) data, and these inputs are processed to find the spatiotemporal patterns present. White boxes indicate data entities; shaded boxes represent analysis methods.

NeuroPatt follows the workflow shown in Fig 11 and includes data filtering to extract oscillatory activity; optical flow estimation to quantify the direction and speed of propagations by constructing velocity vector fields; turbulence-inspired classification and tracking of simple waves (synchrony, planar travelling waves) and complex patterns (sources, sinks, spiral waves, saddles); and vector field decompositions to find dominant spatiotemporal dynamics.

### Oscillatory data filtering

NeuroPatt includes two complementary methods to band-pass filter oscillatory neural data to extract amplitude or phase at a chosen frequency prior to detection of spatiotemporal patterns. The first method uses an eighth-order Butterworth filter as implemented in MATLAB’s Signal Processing Toolbox to filter data to a specified frequency range. This filter is applied in both forward and reverse directions to minimize phase distortion. The oscillation amplitude, *A*, and phase, *θ*, are then extracted from the analytic signal, $z_{f}+i{\hat{z}}_{f}=Ae^{i\theta }$, where ${\hat{z}}_{f}$ is the Hilbert transform [39] of the filtered data $z_{f}$. The second method estimates the analytic signal at a specified center frequency using the complex Morlet wavelet transform to filter data and extract phase and amplitude with an optimal trade-off between time and frequency resolution [40], as implemented by MATLAB’s Wavelet Toolbox.

These two procedures give comparable outputs [67], but each has advantages in different situations: The Hilbert transform allows the properties of the filtering to be precisely specified but can be invalid if the underlying frequency is not sufficiently narrow-band; the wavelet transform is usually faster to compute and always results in a valid analytic signal, but gives a less concretely defined frequency range. Both procedures are included in the toolbox, with the wavelet transform as the default option. Users without access to either the Signal Processing Toolbox or the Wavelet Toolbox can detect spatiotemporal patterns in unfiltered data, which are valid but can be contaminated by noise from multiple frequencies as illustrated in Fig 8, or can calculate the analytic signal through other implementations of the Hilbert or wavelet transform for use in the later steps of the toolbox. Any band-pass filtering procedure necessarily involves some degree of temporal smoothing [68], which can inhibit the extraction of precise timing information in later analysis steps. We note that this effect will not change the timing of maxima or minima in time series, as both wavelet and Hilbert filtering techniques do not distort signal phase, but they will smear out activity between these points.

### Solving optical flow equations

Velocity vector fields are calculated by solving the Euler-Lagrange equations corresponding to the minimization problem given by Eq 4:

$$\rho^{\prime }\left(E_{d}^{2}\right)D_{x}\left[D_{x}u+D_{y}v+D_{t}\right]-\alpha \nabla \cdot \left[\rho^{\prime }\left(E_{s}^{2}\right)\nabla u\right]=0,$$

$$\rho^{\prime }\left(E_{d}^{2}\right)D_{y}\left[D_{x}u+D_{y}v+D_{t}\right]-\alpha \nabla \cdot \left[\rho^{\prime }\left(E_{s}^{2}\right)\nabla v\right]=0,$$ (10)

Where $\rho^{\prime }\left(x^{2}\right)={\left(2\sqrt{x^{2}+\beta^{2}}\right)}^{-1}$. Note that for large values of *β*, $\rho^{'}\left(x^{2}\right)$ is approximately constant, and the optical flow for the Charbonnier penalty approaches that of the quadratic penalty. For clarity, we let $\rho_{d}=\frac{1}{\alpha }\rho^{\prime }\left(E_{d}^{2}\right)$ and $\rho_{s}=\rho^{\prime }\left(E_{s}^{2}\right)$, and rewrite these equations as

$$\rho_{\text{d}}{\text{D}}_{\text{x}}\left[{\text{D}}_{\text{x}}\text{u}+{\text{D}}_{\text{y}}\text{v}+{\text{D}}_{\text{t}}\right]-\rho_{\text{s}}\nabla^{2}\text{u}-\nabla \rho_{\text{s}}\cdot \nabla \text{u}=0,$$

$$\rho_{d}D_{y}\left[D_{x}u+D_{y}v+D_{t}\right]-\rho_{s}\nabla^{2}v-\nabla \rho_{s}\cdot \nabla v=0,$$ (11)

where $\nabla^{2}≐\left(\partial_{x}^{2},\;\partial_{y}^{2}\right)$ denotes the Laplace operator. These equations can be solved through fixed point iteration for the functions $\rho_{d}$ and $\rho_{s}$ after linearizing all other terms. In the toolbox, we approximate partial first derivatives with a five-point stencil $\frac{1}{12}\left[-1,8,0,-8,1\right]$ where possible (or with centered or forward differences when close to edges), and approximate the Laplacian with a 2D five-point stencil [69]. If *D* represents phase data, it will contain temporal and spatial discontinuities where phase wraps from -π to +π, which invalidate linear difference stencils taken near these points. Instead, we approximate partial derivatives with centered or forward differences calculated using circular subtraction, $\theta_{1}-\theta_{2}=mod\left(\theta_{1}-\theta_{2}+\pi ,2\pi \right)-\pi$. All figures in this report use parameters α=0.1 and β=10 unless otherwise specified.

### Toolbox input data format restrictions

To create valid velocity vector fields, NeuroPatt has some restrictions on the format and content of input data sets. Firstly, data must be spatially arranged in a 2D square lattice of recording sites. This restriction exists primarily because of the optical flow estimation methods implemented in the toolbox, which assume spatial uniformity to maximize efficiency and accuracy. Alternate optical flow estimation methods exist for 3D data sets sampled volumetrically [70] or from non-uniform surfaces [71], but these implementations require significant modifications to the methodology described here and are much more computationally intensive. Secondly, data must be consistent across multiple recording sites: Because velocity vector fields are computed based on local dynamics, any recording sites with erroneous activity can significantly influence the surrounding velocity vectors. As in previous studies using optical flow for brain recordings [58,72], we recommend that highly noisy data are spatially filtered prior to the application of optical flow methods, and that any invalid or discontinuous recording channels are interpolated over. When amplitude spatiotemporal patterns are being examined, data should also be normalized across recording sites by subtracting the baseline or z-scoring to remove factors that could cause any regional bias, such as uneven electrode impedance or dye intensity. These processes are included as optional pre-processing steps in the toolbox.

Finally, the changes between consecutive time steps in recorded signals must also be sufficiently small, as optical flow cannot be estimated if there are large discontinuities between frames. Such discontinuities can occur if signals are changing on a shorter time scale than the sampling frequency, which may be an issue for fast neural signals such as action potentials and high-frequency oscillations, or for recording techniques with low temporal resolution such as fMRI. There is no strict rule to determine if the sampling frequency is sufficiently high, but as a general guideline we suggest that signals at a single recording site should typically change by less than 10% of their maximum range between consecutive time steps. The toolbox will warn if this condition is not satisfied, potentially leading to non-convergent optical flow estimation or invalid velocity vector fields, or if the change in data is significantly below this threshold, indicating that it can be safely down-sampled for computational efficiency.

### Tracking spatiotemporal patterns

The toolbox includes multiple parameters for the identification and tracking of spatiotemporal patterns. Firstly, the user can specify a minimum distance from the edge of the recorded area $L_{edge}$ (default 2 grid spaces) for critical points to occupy, as velocity fields can be inaccurate and contain spurious critical points close to the boundary (mainly due to the use of forward differences to approximate derivatives at these points). The user can also specify a minimum radius $L_{radius}$ (default 2 grid spaces) for critical point patterns to occupy, to exclude small-scale and potentially noise-driven local patterns from analysis.

Once both simple and complex patterns are detected in individual velocity fields, these individual observations are combined across time to identify persistent spatiotemporal patterns. NeuroPatt allows the user to specify a minimum duration $t_{dur}$ (default 5 time steps) for all patterns (including global synchrony and plane waves): patterns which persist for less than this amount of time are discarded. To add some error tolerance to the process of linking patterns together over time, a maximum time gap $t_{gap}$ (default 1 time step) can be specified between successive instances of a pattern for it to still be counted as one spatiotemporal structure. Finally, complex patterns can move over time, so critical points of the same type in successive time steps are considered part of the same spatiotemporal pattern only if they are separated by less than a maximum displacement $L_{disp}$ (default 0.5 grid spaces).

The pattern detection methods and all relevant parameters can therefore be summarized as follows: Synchrony occurs when $R\left(t\right)>T_{syn}$ at least every $t_{gap}+1$ time steps for a period of at least $t_{dur}$. Plane waves occur when $\varphi \left(t\right)>T_{pw}$ at least every $t_{gap}+1$ time steps for a period of at least $t_{dur}$. Nodes, foci and saddles occur when a critical point, at least $L_{edge}$ away from the edge of the grid with a minimum spatial radius of $L_{radius}$, can be linked to another critical point of the same type within $t_{gap}+1$ time steps and distance $L_{disp}$, and this chain of critical points persists for at least $t_{dur}$ time steps. Nodes and foci with the same stability properties are typically treated as separate patterns. However, they represent the same type of motion (expansion from or contraction to the critical point), so the toolbox can optionally group these critical points together for a more robust characterization of these pattern types.

### Pattern detection parameters and result validation

NeuroPatt contains a few key parameters that must be carefully selected by the user to ensure valid and accurate results. As illustrated in Fig 2, the optical flow smoothness parameter α controls many properties of the computed velocity vector fields and can over-smooth the data and create spurious plane wave activity if too large or generate flow fields dominated by local noise if too small. To assist with the selection of appropriate values of these parameters, the NeuroPatt toolbox can automatically generate simulated datasets with recording size, sampling frequency and oscillation frequency identical to input data, allowing α and β to be optimized based on the user’s data, as shown in Fig 3. Because the pattern dynamics are typically unknown prior to processing they must be guessed for the simulated data, but we find that in most cases the optimal parameter choices do not change significantly with the pattern types or sizes present.

Even within valid velocity fields, the detection of plane waves and synchronized activity is largely dependent upon the thresholds $T_{pw}$ and $T_{syn}$, which are typically arbitrary parameters set by the user. This is a persistent problem in the detection of such activity patterns: Previous studies have identified plane waves using template matching [24,30,73] or though alignment statistics [17]; and synchronous activity through correlation or coherence measures [74,75]. All these methods rely on largely arbitrary thresholds to explicitly detect patterns. To assist with the choice of these thresholds in our methodology, we implement an optional visual inspection step in the toolbox, allowing users to view sample periods of plane wave and synchronous activity for various thresholds and display distributions of the underlying velocity field statistics across a recording. If such distributions are multi-modal, they can suggest meaningful boundary points for threshold values.

To help to validate results, the NeuroPatt toolbox implements surrogate data generation to test results obtained from real data against results obtained from noise with the same basic dynamics as the input data. To achieve this, we construct time series comprising white noise for each recording site with the same mean and standard deviation of the corresponding site in the original data. We then repeat all processing steps including pre-processing, filtering, optical flow estimation and pattern detection with multiple random surrogate datasets and compare identified pattern statistics and dynamics with those obtained from the true data. If results are comparable between the real and surrogate datasets, it suggests that they may have been introduced through smoothing or other processing steps rather than being real effects in the data. This surrogate data testing process will therefore flag most false positive detections made by the toolbox. To ensure that all findings are robust to changes in parameters, we recommend that users verify that their results are consistent across a range of values for key parameters in the pattern detection process.

### Pattern evolution dynamics

Once simple and complex spatiotemporal patterns have been detected, the evolution dynamics between different pattern types can be quantified. For each pair of pattern types ($p_{A},\;p_{B}$), the observed number of transitions from $p_{A}$ to $p_{B},\;n_{obs}\left(p_{A}\to p_{B}\right)$, can be counted in each trial by searching for instances where $p_{B}$ starts within a short time gap $t_{gap}$ of $p_{A}$ ending. This can be compared to the expected number of transitions if the patterns in the trial occurred at random times,

$$n_{exp}\left(p_{A}\to p_{B}\right)=\frac{n_{A}n_{B}t_{gap}}{t_{trial}},$$ (12)

where $n_{A}$ and $n_{B}$ are the observed number of patterns $p_{A}$ and $p_{B}$ within the trial and $t_{trial}$ is the total length of the trial in seconds. The fractional change between observed and expected transition counts is then defined as $n_{obs}/n_{exp}-1$. We used paired t-tests with the Bonferroni correction for multiple comparisons to evaluate whether $n_{obs}$ and $n_{exp}$ were significantly different for each pattern transition across multiple trials of a recording.

### Simulated data

We test the pattern detection procedures in NeuroPatt by generating data sets with known pattern properties. To create a simulated wave pattern *z*_*sim*_ with wavenumber *k* and angular frequency *w*, we use the formula

$$z_{sim}\left(x,y,t\right)=A\left(x,y,t\right)e^{i\left(ks\left(x,y,t\right)-wt\right)},$$ (13)

where *A*(*x*,*y*,*t*) is a function giving the spatial amplitude profile of the wave, and *s*(*x*,*y*,*t*) is a function giving the spatial phase profile. Using different functions for *s* allows different types of critical point patterns to be generated. All patterns are specified with an initial location (*x*_*0*_,*y*_*0*_), and a constant velocity (*v*_*x*_,*v*_*y*_), given in grid spaces per time step, so the location of a pattern at time *t* is $\left(x_{t},y_{t}\right)=(x_{0}+v_{x}t,y_{0}+v_{y}t)$. For source or sink patterns, we use

$$s_{\frac{source}{sink}}\left(x,y,t\right)=\sqrt{{\left(x-x_{t}\right)}^{2}+{\left(y-y_{t}\right)}^{2}},$$ (14)

for spiral patterns we use

$$s_{spiral}\left(x,y,t\right)=\sqrt{{\left(x-x_{t}\right)}^{2}+{\left(y-y_{t}\right)}^{2}}+\frac{1}{k}\text{atan}2\left(y-y_{t},x-x_{t}\right),$$ (15)

where atan2 is the multi-valued inverse tangent, and for saddle patterns we use

$$s_{saddle}\left(x,y,t\right)=\left|x-x_{t}\right|-\left|y-y_{t}\right|.$$ (16)

To ensure that all patterns are localized, we define *A*(*x*,*y*,*t*) as a symmetric two-dimensional Gaussian centered on the critical point location:

$$A\left(x,y,t\right)=A_{0}\exp\left(\frac{-\left({\left(x-x_{t}\right)}^{2}+{\left(y-y_{t}\right)}^{2}\right)}{2c^{2}}\right),$$ (17)

where *A*_*0*_ is the maximum amplitude and *c* is the Gaussian width parameter.

To generate complex datasets, we add multiple wave patterns and then add normally distributed white noise with mean 0 and standard deviation proportional to the amplitude of each grid point. For Fig 3, we used a 12×12×10 spatiotemporal grid to generate datasets comprising two random critical point patterns, both with parameters *w* = 2π×0.01 and *k* = 2π/5. Start positions, velocities, maximum amplitudes and Gaussian width parameters were randomized in each dataset: *x*_*0*_ and *y*_*0*_ were picked uniformly randomly but rejected if patterns were within 2 grid spaces of each other or an edge, *v*_*x*_ and *v*_*y*_ were picked randomly between −0.1 and +0.1, *A*_*0*_ was picked between 1 and 2 and *c* was picked between 3 and 5. Pattern detection in simulated data was performed with default parameters.

### Experimental recordings

To demonstrate methods in NeuroPatt, we analyze recordings from the middle temporal area of adult male marmosets (Callithrix jacchus). Details of preparation are given previously [49,76]. Anesthesia and analgesia were maintained by intravenous infusion of sufentanil citrate (6–30 μg kg^−1^ h^−1^) and inspired 70:30 mix of N_2_O and carbogen (5% CO_2_, 95% O_2_). Dominance of low frequencies (1–5 Hz) in the EEG recording and absence of EEG or electrocardiogram changes under noxious stimulus (tail pinch) were taken as the chief signs of an adequate level of anesthesia. Drifts towards higher frequencies (5–10 Hz) in the EEG record were counteracted by increasing the rate of venous infusion or the concentration of anesthetic. The typical duration of a recording session was 48–72 h.

Stimuli were presented on a cathode-ray-tube monitor (Sony G500, refreshed at 100 Hz, viewing distance 45 cm, mean luminance 45–55 cd m^−2^), and comprised either a grey screen held at constant luminance for the duration of the recording (5–25 minutes, ongoing activity), or a pattern alternating every two seconds between a grey screen and a field of drifting circular white dots (Weber contrast 1.0; dot diameter 0.4°; drift velocity 20 deg/s) presented in a large, stationary circular window (30°). For dot fields, different motion directions (90° steps) were presented pseudo-randomly, and the procedure was repeated until 100 repetitions were made for each of the four directions. Data were recorded using multielectrode arrays (10×10 electrodes, 1.5 mm length, electrode spacing 400 μm, Blackrock Microsystems). Recording surface insertion depth was targeted to 1 mm.

To demonstrate pattern dynamics at a larger spatial scale and during waking activity, we also examine optical voltage recordings from awake mice, obtained with permission from Thomas Knöpfel. Details of recording have been described previously [50,77,78]. Briefly, excitatory neurons in mouse layer 2/3 were targeted with the gene encoding VSFS Butterfly 1.2 [79], and mice were implanted with a head post and thinned skull cranial window. Image acquisition was performed with a dual emission wide-field epifluorescence macroscope during anesthesia induced by pentobarbital sodium (40 mg/kg i.p.). The data presented here were taken as anesthesia was wearing off, when mice were responsive to touch and exhibited spontaneous whisker and limb movement between recordings. Image sequences of 60 s duration were acquired at 50 Hz temporal resolution and 320 × 240 pixel spatial resolution, with each pixel corresponding to 33 × 33 μm of a projected cortical area. The voltage imaging signal was calculated as the ratio of mKate2 to mCitrine fluorescence after equalization of heartbeat-related fluorescence modulation. The resulting ratiometric sequences of voltage maps were then spatially down-sampled by a factor of 5 using the MATLAB function *imfilter*, to smooth over noise and reduce the density of calculated velocity fields.
